# Lipid Composition, Fatty Acids and Sterols in the Seaweeds *Ulva armoricana*, and *Solieria chordalis* from Brittany (France): An Analysis from Nutritional, Chemotaxonomic, and Antiproliferative Activity Perspectives

**DOI:** 10.3390/md13095606

**Published:** 2015-09-02

**Authors:** Melha Kendel, Gaëtane Wielgosz-Collin, Samuel Bertrand, Christos Roussakis, Nathalie Bourgougnon, Gilles Bedoux

**Affiliations:** 1 University of South Brittany, EA 3884, LBCM, IUEM, F-56000 Vannes, France; E-Mails: melha.kendel@gmail.com (M.K.); nathalie.bourgougnon@univ-ubs.fr (N.B.); 2 Faculté des Sciences Pharmaceutiques et Biologiques, LUNAM, Université de Nantes, Groupe Mer-Molécules-Santé MMS, EA 2160, Institut Universitaire Mer et Littoral FR3473 CNRS, 9 Rue Bias, BP 53508, F-44035 Nantes Cedex 1, France; E-Mails: wielgosz-collin@univ-nantes.fr (G.W.-C.); samuel.bertrand@univ-nantes.fr (S.B.); 3 Faculté des Sciences Pharmaceutiques et Biologiques, LUNAM Université, Université de Nantes, IICIMED/ERATU-EA 1155 Cancer du Poumon et Cibles Moléculaires, 1 Rue Gaston Veil, BP 53508, F-44035 Nantes Cedex 01, France; E-Mail: christos.roussakis@univ-nantes.fr

**Keywords:** *Ulva armoricana*, *Solieria chordalis*, fatty acids, sterols, phospholipids, glycolipids, polyunsaturated fatty acids, hydroxy fatty acids, chemotaxonomy, human lung cancer

## Abstract

Lipids from the proliferative macroalgae *Ulva* *armoricana* (Chlorophyta) and *Solieria chordalis* (Rhodophyta) from Brittany, France, were investigated. The total content of lipids was 2.6% and 3.0% dry weight for *U. armoricana* and *S. chordalis*, respectively. The main fractions of *S. chordalis* were neutral lipids (37%) and glycolipids (38%), whereas *U. armoricana* contained mostly neutral lipids (55%). Polyunsaturated fatty acids (PUFA) represented 29% and 15% of the total lipids in *U. armoricana* and *S. chordalis*, respectively. In both studied algae, the phospholipids were composed of PUFA for 18%. In addition, PUFA were shown to represent 9% and 4.5% of glycolipids in *U. armoricana* and *S. chordalis*, respectively. The essential PUFA were 16:4*n*-3, 18:4*n*-3, 18:2*n*-3, 18:2*n*-6, and 22:6*n*-3 in *U. armoricana*, and 20:4*n*-6 and 20:5*n*-3 in *S. chordalis*. It is important to notice that six 2-hydroxy-, three 3-hydroxy-, and two monounsaturated hydroxy fatty acids were also identified and may provide a chemotaxonomic basis for algae. These seaweeds contained interesting compounds such as squalene, α-tocopherol, cholest-4-en-3-one and phytosterols. The antiproliferative effect was evaluated *in vitro* on human non-small-cell bronchopulmonary *carcinoma* line (NSCLC-N6) with an IC_50_ of 23 μg/mL for monogalactosyldiacylglycerols isolated from *S. chordalis* and 24 μg/mL for digalactosyldiacylglycerols from *U. armoricana*. These results confirm the potentialities of valorization of these two species in the fields of health, nutrition and chemotaxonomy.

## 1. Introduction

Seaweeds are known for their richness in nutritionally beneficial components and contain bioactive compounds such as proteins, carbohydrates, antioxidants, minerals, dietary fibers, vitamins and polyunsaturated fatty acids (PUFAs) [1].

In recent years, lipid composition in marine algae has raised considerable interest due to their high content of PUFAs, specifically α-linolenic (18:3*n*-3), octadecatetraenoic (18:4*n*-3), arachidonic (20:4*n*-6), and eicosapentaenoic acids (20:5*n*-3) [2]. This class of acids was considered as essential nutritional components in humans and animals [3]. For example, they play an important role in the prevention of cardiovascular diseases, osteoarthritis, diabetes, and it possesses antimicrobial, antiviral, anti-inflammatory and antitumoral properties [2,3].

Furthermore, studies dealing with fatty acids (FA) of seaweeds are also interesting for the usefulness of FA as potential chemotaxonomic biomarkers. Their lipid profiles could assist the assignment of algal taxonomic position and provide signature profiles for use in organic geochemistry and food studies [4]. Such biochemical analysis may also be useful to study the abundance and ecology of these species in the marine environments.

An attractive approach to find sources of marine lipids, could be the exploitation of coastal proliferative seaweeds species especially if they contain interesting substances. This is the case for *Solieria chordalis* (Rhodophyta) and for *Ulva armoricana* (Chlorophyta), which are proliferative species found in Brittany (North West of France).

Thus, the present study explores the lipid composition of *S. chordalis* for the first time and of *U. armoricana* for extending the knowledge on its lipid composition where little is known about FA composition of individual lipid classes. The aim is the determination of the potential value of lipid composition (total lipid, glycolipid—GL, phospholipids—PL, sterol and unsaponifiable fraction) of these proliferative species, which may constitute a nutritional database for chemotaxonomy and anti-proliferative activity perspectives.

So, for these two species, the present study was designed to clarify the content of lipid and glycolipid (GL) classes, and the FA composition from total lipids, phospholipids (PL), GL and compounds of unsaponifiable fraction. In addition, the GL fractions were evaluated for their anti-proliferative activity against human cancer cell lines (non-small cell lung cancer-NSCLC-N6). This present study particularly focuses on lung cancer and to the best of our knowledge for the first time for these species, lung cancer being the leading cause of cancer-related mortality in both men and women in the world.

## 2. Results and Discussion

### 2.1. Lipid Contents and Lipid Classes Distribution

The specimens of Chlorophyta (*Ulva armoricana*) and Rhodophyta (*Solieria chordalis*) used in the present investigation were based on their wide distribution and abundance in Brittany (North West of France). The total lipid (TL) contents and the lipid class distribution are reported in Table 1.

**Table 1:** Total lipid contents (% dry weight) and lipid class distribution (% total lipids) of *U. armoricana* and *S. chordalis.*

	*U. armoricana* (%)	*S. chordalis* (%)
Water content fresh algae	92.00 ± 1.25	90.05 ± 0.70
Total lipids	2.62 ± 0.04	2.96 ± 0.04
Neutral lipids	55.60 ± 0.05	37.70 ± 0.07
Glycolipids	29.10 ± 0.10	38.50 ± 0.02
Phospholipids	15.30 ± 0.10	23.70 ± 0.09

Values are the mean of three replicates (mean (% dw) ± s.d.); s.d., standard deviation.

The TL content of *U. armoricana* and *S. chordalis* was established at 2.62% ± 0.04% dry weight (dw) and 2.96% ± 0.04% (dw), respectively. These contents are in agreement with the literature [5,6]. Table 2 shows the TL contents previously found in various *U. armoricana* ranging from 0.2% to 7.9% dw and for *Solieria* sp. whose levels vary from 0.4% to 2.8%. The values determined in this study are higher than those reported for different species of the same genus, as shown in Table 2. Nevertheless, it is interesting to note that brown seaweeds generally contain higher TL levels [5]. Furthermore, the TL contents of *U. armoricana* and *S. chordalis* are lower compared to earth vegetables, such as soy or sunflower [6].

**Table 2:** Total lipid contents of various algal *Ulva* and *Solieria* species.

Phylum	Algal Species	Location	Date	Reported Levels (%)	References
Chlorophyta	*Ulva* sp.	Beach in Plestin-les-Grèves, France	June 2012	0.6% dw	[7]
		north-east part of the Black Sea (Feodosiya and Karadag Bays, Crimea)	July 1987	0.2% dw	[8]
	*U. lactuca*	Coast of Abu Qir Bay near Boughaz El-Maadya (Egypt)	April, August and October 2010	4.0% dw	[9]
		Buleji beach of Karachi coast	Different seasons at low tide	1.2% dw	[10]
		Littoral between the area of Téboulba and Sayada (Monastir-Tunisia)	July 2007	7.9% dw	[11]
Chlorophyta	*U. lactuca*	Indian Sundarbans	September 2007–June 2008	0.3%–1.0% dw	[12]
		Bohai Sea near Weihai (China)	August	1.2% fw	[13]
		Bodega Bay, California	November 1995	3.1% dw	[14]
		Coast of Norway	May and June 2012	2.6% dw	[15]
		Coast of the Persian Gulf (Iran)	-	3.6% dw	[16]
		Ria de Aveiro	October 2012	0.3% dw	[17]
	*U. rigida*	north-east part of the Black Sea (Feodosiya and Karadag Bays, Crimea)	July 1987	0.3% dw	[8]
		Varvara, Bulgaria	October 2011	0.8% fw	[18]
	*U. fenestrata*	Pacific Ocean in the Northern part of Canada	-	0.5% la	[19]
	*U. pertusa*	Bohai Sea near Weihai (China)	August	0.9% fw	[13]
	*U. lactuca* *U. fasciata* *U. taeniata* *U. pertusa* *U. reticulata* *U. beytensis* *U. compressa* *U. rIgida* *U. linza* *U. flexuosa* *U. erecta* *U. prolifera*	Gujarat coast, India	March–October 2011	0.7%–2.0% fw	[20]
	*U. linza*	Abu Qir Bay, Egypt	Spring, summer, autumn	4.1% dw, 3.8% dw, 3.2% dw	[21]
Rhodophyta	*S. chordalis*	Littoral zone of the Saint Gildas de Rhuys, France	March 2012	0.9% dw	[7]
	*S. robusta*	Gujarat coast, India	March–October 2011	0.4%–1.0% fw 0.9% fw	[20]
		Buleji beach of Karachi coast	Different seasons at low tide	2.8% dw	[10]
		Sea of South China	-	0.3% la	[19]

dr, dry weight; fw, fresh weight; la, lyophilized algae.

These variations could be related to taxonomic entity, seasonality of sampling, location and macroalgae growth conditions [22], in addition to extraction processing and solvent polarity [23]. Sánchez-Machado *et al.* reported that, as the temperature increased, the lipid level decreased and remained almost stable until the end of the growing season [24]. In comparison to *Grateloupia turuturu* (Halymeniaceae), the lipid contents are lower, *G. turuturu* TL content being 3%–4% dw [25]. Each of the TL extracts was fractionated into three fractions corresponding to lipid classes (Table 1): neutral lipids (NL—storage lipids), glycolipids (GL) and phospholipids (PL—structural lipids). For *S. chordalis*, the major lipid classes corresponded to NL and GL, accounting for 38% each, followed by PL (24%). In the case of *U. armoricana*, the main lipid class was NL with 56%, followed by GL (29%) and by PL (15%).

### 2.2. Lipid Composition

#### 2.2.1. Neutral Lipid and Sterol Composition

*U. armoricana* and *S. chordalis* were examined for their hydrocarbons and sterols present in unsaponifiable fractions. The analyses were carried out by gas chromatography coupled to mass spectrometry (GC-MS) and observed compositions are given in Table 3.

**Table 3:** Composition (%) of the unsaponifiable fractions of *U. armoricana* and *S. chordalis.*

	*U. armoricana* (%) (21% Total Lipids)	*S. chordalis* (%) (10% Total Lipids)
**Hydrocarbons**		
Hydrocarbons	4.3 ± 0.1	5.7 ± 0.2
Squalene	2.7 ± 0.1	4.5 ± 0.1
**Total hydrocarbons**	**7.0 ± 0.1**	**10.2 ± 0.1**
**Sterols**		
22(*E*)-Dehydrocholesterol	1.0 ± 0.1	1.3 ± 0.1
Cholesterol	35.2 ± 0.3	42.6 ± 0.2
Campesterol	1.3 ± 0.1	1.7 ± 0.1
Brassicasterol	3.0 ± 0.2	2.1 ± 0.1
22-Dehydrolathosterol	0.8 ± 0.1	1.0 ± 0.1
Fucosterol	1.4 ± 0.1	2.3 ± 0.1
Isofucosterol	25.0 ± 0.2	nd ^a^
Cholest-4-en-3-one	0.8 ± 0.1	2.0 ± 0.1
**Total sterols**	**68.5 ± 0.1**	**53.0 ± 0.1**
**Other compounds**		
α-Tocopherol	5.5 ± 0.1	3.1 ± 0.1
Phytol	19.0 ± 0.1	33.7 ± 0.3

Values are the mean of three replicates (mean (% dw) ± s.d.); s.d., standard deviation; ^a^ nd, not detected.

Hydrocarbons represented 7% of the unsaponifiable fraction for *U. armoricana*, among which squalene corresponded to 2.7% (Table 3). It is interesting to note that a rich squalene diet enhances anti-tumor activity of some chemotherapeutic agents by increasing immune system efficiency and by lowering blood cholesterol content [26]. Moreover, there is some evidence that squalene reduces colon cancer and skin cancer; this activity likely being related to its antioxidant status [27]. Therefore, squalene may be beneficial in preventive therapy and integrative medicine. Additionally, α-tocopherol represented 5.5% of the unsaponifiable fraction from *U. armoricana* (Table 3), and is an important natural antioxydant [28].

Moreover, it also contained 19% phytol (Table 3). This particular compound is usually used as a precursor for the industrial synthesis of vitamins E and K [29,30].

The major constituent of the unsaponifiable fraction from *U. armoricana* was cholesterol (35%), which is one of the main sterols present in seaweeds. Its content in green and brown algae varies from 2% to 76% of total sterol. In red algae, its content is lower; however, it is still among the major components of the sterol fraction [31]. Some green algae, such as *Ulva* and *Chaetomorpha*, contain cholesterol or 28-isofucosterol as their principal sterol [32].

Cholest-4-en-3-one was also detected as a minor component for *U. armoricana* (0.8%, Table 3). This compound is a key intermediate in steroid chemistry, which is known as a cholesterol derivative occurring in both plant and animal tissues [33]. It may result from the biosynthesis or the autoxidation of cholesterol. Cholest-4-en-3-one, known as an intestinal catabolite of cholesterol, has an anti-obesity effect on animals [34].

Finally, the unsaponifiable fraction of *U. armoricana* also contained other phytosterols. They corresponded to campesterol, brassicasterol, isofucosterol, which are known to exhibit cholesterol-lowering effects by decreasing intestinal cholesterol absorption [35]. Furthermore, the activity of phytosterols on cardiovascular diseases and their potent anti-inflammatory properties have been reported [35]. For Humans, all phytosterols come from dietary sources, as Humans cannot *de novo* synthesize phytosterols.

Aknin *et al.* have investigated sterol composition of three Chlorophyceae orders and showed that sterol composition offers distinguishing features for the chemotaxonomic classification of these algae [36]. Isofucosterol is typical for Ulotrichales and particularly the Ulvaceae family. Nobuo *et al.* have also found that the sterol content in marine Chlorophyta is similar to higher plants [37].

Regarding *S. chordalis*, hydrocarbons represented 10.2% of the unsaponifiable fraction, among which squalene corresponded to 4.5%. Furthermore, α-tocopherol and phytol were also detected and represented 3.1% and 34% of the unsaponifiable fraction, respectively. The main component of the unsaponifiable fraction was cholesterol (43%). Other minor sterols were detected such as cholest-4-en-3-one, but no isofucosterol was detected (Table 3).

Our results about sterols composition of *S. chordalis* (Gigartinales) showed differences with the results of Nasir *et al.* who identified 22-dehydrocholesterol, cholesterol and stigmasterol from *Gracilaria salicornia* (Gigartinales), and 22-dehydrocholesterol, cholesterol, cholesterol oleate, and (22*E*)-cholesta-5,22-dien-3β-ol-7-one from *Hypnea flagelliformis* (Gigartinales) [38].

#### 2.2.2. Fatty Acid Composition of Total Lipids

All fatty acids (FAs) of the TL were converted into the fatty acid methyl esters (FAMEs) by transmethylation with methanolic hydrogen chloride. The FAs were identified as FAME by comparing their equivalent chain lengths (ECLs) values with those previously described or by using commercial mixtures. The ECLs of the FAMEs were determined by expressing their elution positions relative to those of known straight-chain saturated FAMEs.

FAMEs were converted to *N*-acyl pyrrolidides (NAPs) in order to locate double bonds and branching [28]. The FA composition of TL is given in Table 4.

**Table 4:** Fatty acid (% of the total FAs mixture) of *U. armoricana* and *S. chordalis.*

Fatty Acid (FAs) ^a^	ECL ^c^	*U. armoricana* (%)	*S. chordalis* (%)
**Saturated fatty acids (SFAs)**			
14:0	14.00	0.6 ± 0.2	2.6 ± 0.2
15:0	15.00	0.4 ± 0.1	1.4 ± 0.1
16:0	16.00	42.0 ± 0.2	45.0 ± 0.2
18:0	18.00	1.0 ± 0.1	9.9 ± 0.1
22:0	22.00	2.3 ± 0.1	nd ^b^
24:0	24.00	0.2 ± 0.1	nd ^b^
**Total SFAs**		**46.5 ± 0.1**	**58.9 ± 0.1**
**Monounsaturated fatty acids (MUFAs)**			
14:1	13.63	0.4 ± 0.1	nd ^b^
15:1	14.57	nd ^b^	0.2 ± 0.1
16:1*n*-9	15.79	2.7 ± 0.1	nd ^b^
16:1*n*-7	15.94	1.9 ± 0.1	nd ^b^
16:1	15.76	nd ^b^	0.2 ± 0.1
17:1*n*-7	16.77	nd ^b^	18.5 ± 0.1
18:1	17.14	1.9 ± 0.1	nd ^b^
18:1*n*-9	17.71	nd ^b^	3.4 ± 0.1
18:1*n*-7	17.78	17.3 ± 0.1	4.0 ± 0.1
**Total MUFAs**		**24.3 ± 0.1**	**26.3 ± 0.1**
**Polyunsaturated fatty acids (PUFAs)**			
14:2	13.53	1.6 ± 0.1	nd ^b^
16:4*n*-3	15.61	6.4 ± 0.1	nd ^b^
18:4*n*-3	17.70	8.6 ± 0.1	nd ^b^
18:3*n*-3	17.77	0.5 ± 0.1	nd ^b^
18:2*n*-6	17.80	3.7 ± 0.1	nd ^b^
18:2*n*-3	17.87	8.4 ± 0.1	nd ^b^
20:5*n*-3	19.21	Trace	5.0 ± 0.1
20:4*n*-6	19.33	Trace	9.8 ± 0.1
**Total PUFAs**		**29.2 ± 0.1**	**14.8 ± 0.1**
**Total *n*-6 PUFAs**		**3.7 ± 0.1**	**9.8 ± 0.1**
**Total *n*-3 PUFAs**		**23.9 ± 0.1**	**5.0 ± 0.1**
**Ratio *n*-6/*n*-3**		**0.1 ± 0.1**	**1.9 ± 0.1**

^a^ Methyl ester (%) of total FA of total FA mixture; ^b^ nd, not detected; ^c^ ECLs, equivalent chain lengths; Minor FA (≤0.1%), Docosenoic (22:1); Values are the mean of three replicates (mean (% dw) ± s.d.); s.d., standard deviation.

The saturated fatty acids (SFAs) corresponded to 46.5% and 58.9% of TL from *U. armoricana* and *S. chordalis*, respectively. Their chain length ranged from C_14_ to C_24_ for *U. armoricana* and from C_14_ to C_18_ for *S. chordalis*. In the case of *U. armoricana*, the major SFAs were palmitic acid (42%) and behenic acid (2.3%). For *S. chordalis*, the major SFA were palmitic acid (45%) and stearic acid (10%). Monounsaturated FAs (MUFAs) contents were 24.3% for *U. armoricana* and 26.3% for *S. chordalis*. The predominant MUFAs were 16:1*n-*9 (2.7%) and 18:1*n-*7 (17.3%) for *U. armoricana*, and were 17:1*n-*7 (18.5%), 18:1*n-*7 (4%) and 18:1*n-*9 (3.4%) for *S. chordalis*.

PUFAs corresponded to 29.2% and 14.8% of the TL composition of *U. armoricana* and *S. chordalis*, respectively. The main PUFAs observed in *U. armoricana* TL were 16:4*n-*3 (6.4%), 18:4*n-*3 (8.6%), 18:2*n-*6 (3.7%) and 18:2*n-*3 (8.4%), with traces of arachidonic 20:4*n-*6 (AA), eicosapentaenoic 20:5*n-*3 (EPA), and docosahexaenoic 22:6*n-*3 (DHA) acids. In the case of *S. chordalis*, 20:4*n-*6 (9.8%) and 20:5*n-*3 (5%) acids were the major PUFAs.

These results are in agreement with the literature concerning seaweeds. Red seaweed species contain significant quantities of PUFA, up to 20 carbons, with four or five double bonds. Their two major PUFAs are AA and EPA. In addition, a high C_20_/C_18_ PUFA ratio is observed with high C18:1 content [20]. Green seaweeds are characterized by C_16_ and C_18_ PUFA with a high C_18_/C_20_ PUFA ratio [14].

According to the literature, palmitic acid (16:0) is predominant in seaweeds [39]. As in the vegetative tissues of higher plants, the green algae contain primarily C_16_ and C_18_ fatty acids with a high degree of unsaturation [32]. The amount of 16:4*n-*3 varies from 4.9% to 23.4%, which is characteristic of green algae in addition with the presence of 16:3 [40]. Unlike red and brown algae, green algae contain large amounts of 16:3 and 16:4 PUFAs [40]. Johns *et al.* have proposed that 16:4 can be taxonomically characteristic of green macrophytic algae [41], as in plants of other divisions, it is only found in trace amount. Green algae are characterized by high contents of 18:2 and 18:3, like in land plants, *U. armoricana* being very rich in C_18_ PUFAs [13,42]. Linoleic acid (18:2*n-*6) is the main PUFA of most chlorophytes [13,14] and the α-linolenic acid (18:3*n-*3) is characteristic of the Ulvales [13,14].

Furthermore, marine algae also contain the *n-*3 PUFAs. The *n-*3 long-chain PUFAs are abundant in most red algae, and this is similar for the *n-*6 long-chain PUFAs and 18:4*n-*3 [43].

In comparison with other species, the FA profiles obtained for *S. chordalis* were consistent with those reported in previous studies [13,19,20,22,25,43,44].

According to the literature, the PUFAs 20:4*n-*6 and 20:5*n-*3 are predominant in marine red algae, such as *S. robusta* and *S. chordalis* [13]. Furthermore, according to Aknin *et al.* [44] who reported the FAs composition of five Solieriaceae, almost 40 fatty acids were identified, the major fatty acids being 16:0, 16:1*n-*5, 4-OH-3,5-diiodo phenyl (dihydrohydnocarpic), 18:1*n-*9, 18:1*n-*7, 14:0, dihydrochaulmoogric, 18:1*n-*5, 20:4*n-*6 and 20:5*n-*3.

Although *U. armoricana* and *S. chordalis* displayed high amounts of SFA, the contents of PUFAs ranged from 14.8% to 29.2%. It confirms that seaweeds contain significantly higher levels of PUFAs than land vegetables [6]. Interestingly, marine algae are rich in PUFA of the *ω*3 and *ω*6 series, which are considered essential FA. The World Health Organization currently recommends that the *n-*6/*n-*3 ratio should not exceed 10 in a diet [45]. Therefore, *U. armoricana* and *S. chordalis* may be used for the reduction of the *n-*6/*n-*3 ratio, as in TL, the *n-*3 PUFA of *U. armoricana* (23.9%) is higher than the *n-*6 PUFA (3.7%) and the *n-*3 PUFA of *S. chordalis* (5%) is lower than the *n-*6 PUFA (9.8%). The *n-*6/*n-*3 ratio was established at 0.1 for *U. armoricana* and 1.9 for *S. chordalis*.

In addition, the benefits of PUFAs in human health are well documented, including cardiovascular effects [46]. Particularly, the *n-*3 PUFA may be beneficial for the prevention of several types of cancer, and exhibits various biological activities such as decrease of blood pressure and improvement of heart and liver function in body fat in animal trials [47]. Generally, the marine *n-*3 PUFAs exert anti-arteriosclerosis, anti-hypertensive, anti-inflammatory, immune-regulatory, antioxidant and anti-thrombotic effects, and antiarrhythmic responses [48]. In addition, they are precursors of the eicosanoids biosynthesis, which are bioregulators in many cellular processes [39]. The impact of *n-*3 PUFAs on brain function and mental health has also been recently examined, showing that they are able to improve the mitochondrial function [49]. Both AA and EPA are precursors of prostaglandins, thromboxane and other eicosanoids, which influence inflammation processes and immune reactions [47]. Linoleic acid, linolenic acid and arachidonic acid serve important functions in skin growth and protection. Finally, 20:4*n-*6, 20:5*n-*3, and 20:3*n-*6 lipids have valuable biological activities such as heart and mental health, arthritis, cancer and lung disease [13].

#### 2.2.3. Fatty Acid Composition of Phospholipids

The PL FAs of *U. armoricana* and *S. chordalis* were identified by GC-MS analyses as FAMEs and *N*-acyl pyrrolidides (NAPs) as previously described for FAs of the TL. For many FAs, GC-MS data of the NAP derivatives allowed us to confirm their structures and to determine the location of double bonds, branching and hydroxyl groups [50].

More than 35 FAs were identified in PL of *U. armoricana* and 19 FAs in PL of *S. chordalis* as showed in Table 5.

**Table 5:** Fatty acid (% of the phospholipids FAs mixture) of *U. armoricana* and *S. chordalis*.

Fatty Acid (FAs) ^a^	ECL ^c^	*U. armoricana* (%)	*S. chordalis* (%)
**Saturated fatty acids (SFAs)**			
13:0	13.00	1.7 ± 0.2	nd ^b^
14:0	14.00	0.9 ± 0.1	2.3 ± 0.1
15:0	15.00	0.2 ± 0.2	0.4 ± 0.1
16:0	16.00	53.6 ± 0.1	29.0 ± 0.1
17:0	17.00	0.4 ± 0.1	0.8 ± 0.1
18:0	18.00	4.6 ± 0.1	5.0 ± 0.1
19:0	19.00	0.2 ± 0.0	nd ^b^
20:0	20.00	0.3 ± 0.1	nd ^b^
21:0	21.00	0.2 ± 0.0	nd ^b^
22:0	22.00	1.9 ± 0.2	nd ^b^
24:0	24.00	0.3 ± 0.1	nd ^b^
**Total SFAs**		**64.3 ± 0.1**	**37.5 ± 0.1**
**Monounsaturated fatty acids (MUFAs)**			
15:1	14.57	0.2 ± 0.1	nd ^b^
16:1*n*-11	15.70	1.7 ± 0.1	nd ^b^
16:1*n*-8	15.85	nd ^b^	3.0 ± 0.1
16:1*n*-7	15.94	1.2 ± 0.1	nd ^b^
17:1*n*-14	16.73	3.6 ± 0.1	nd ^b^
17:1*n*-7	16.77	nd ^b^	8.6 ± 0.1
17:1*n*-4	16.88	nd ^b^	10.2 ± 0.2
18:1*n*-9	17.71	nd ^b^	2.2 ± 0.2
18:1*n*-7	17.78	5.8 ± 0.1	3.2 ± 0.1
18:1*n*-3	17.96	nd ^b^	9.6 ± 0.1
19:1	18.54	0.5 ± 0.2	nd ^b^
22:1	21.60	0.4 ± 0.1	nd ^b^
**Total MUFAs**		**13.4 ± 0.1**	**36.8 ± 0.1**
**Polyunsaturated fatty acids (PUFAs)**			
16:4*n*-3	15.60	2.2 ± 0.1	nd ^b^
18:4*n*-3	17.70	3.3 ± 0.1	nd ^b^
18:3	17.75	nd ^b^	1.2 ± 0.2
18:3*n*-3	17.77	7.6 ± 0.2	nd ^b^
18:2	17.79	nd ^b^	2.1 ± 0.1
18:2*n*-6	17.80	1.7 ± 0.2	nd ^b^
20:5*n*-3	19.21	0.4 ± 0.1	2.6 ± 0.1
20:4*n*-6	19.32	0.4 ± 0.1	12.2 ± 0.1
20:3*n*-9	19.44	0.4 ±0.1	nd ^b^
20:2	19.56	0.4 ± 0.1	nd ^b^
21:3	20.64	0.4 ± 0.1	nd ^b^
22:6*n*-3	21.54	0.3 ± 0.1	nd ^b^
22:5	21.63	0.8 ± 0.1	nd ^b^
22:3	21.83	0.6 ± 0.1	nd ^b^
**Total PUFAs**		**18.5 ± 0.1**	**18.1 ± 0.1**
**Hydroxy fatty acids (Hydroxy FAs)**			
**2-Hydroxy FAs**			
2-OH-16:1	17.18	nd ^b^	1.5 ± 0.1
2-OH-16:0	17.31	1.0 ± 0.1	1.4 ± 0.1
2-OH-17:0	18.26	0.2 ± 0.1	4.5 ± 0.1
2-OH-18:0	19.24	1.0 ± 0.1	nd ^b^
2-OH-22:0	23.26	0.1± 0.1	nd ^b^
**3-Hydroxy FAs**			
3-OH-17:1	18.53	0.2± 0.1	nd ^b^
**Total Hydroxy FAs**		**2.5 ± 0.1**	**7.4 ± 0.1**
**Total Fatty aldehyde**		**1.3 ± 0.1**	**nd ^b^**
**dimethylacetals**			
**Total *n*-6 PUFAs**		**2.1 ± 0.1**	**12.2 ± 0.1**
**Total *n*-3 PUFAs**		**13.8 ± 0.1**	**12.2 ± 0.1**
**Ratio *n*-6/*n*-3**		**0.1 ± 0.1**	**1.0 ± 0.1**

^a^ Methyl ester (%) of total FA of total FA of total PL; ^b^ nd, not detected; ^c^ ECLs, equivalent chain lengths; Values are the mean of three replicates (mean (% dw) ± s.d.); s.d., standard deviation.

The SFAs represented 64.3% and 37.5% of the PL from *U. armoricana* and *S. chordalis*, respectively. They ranged from C_13_ to C_24_ for *U. armoricana* and from C_14_ to C_18_ for *S. chordalis*. The major SFAs for *U. armoricana* were palmitic acid (53.6%), and stearic acid (4.6%). For *S. chordalis*, the main SFAs were myristic acid (2.3%), palmitic acid (29%) and stearic acid (5%).

MUFAs content represented 13.4% and 36.8% of PL fraction for *U. armoricana* and *S. chordalis*, respectively. The main MUFAs of *U. armoricana* were 17:1*n-*14 (3.6%) and 18:1*n-*7 (5.8%). In the case of *S. chordalis*, the major MUFAs were 16:1*n-*8 (3%), 17:1*n-*7 (8.6%), 17:1*n-*4 (10.2%), 18:1*n-*7 (3.2%) and 18:1*n-*3 (9.6%).

PUFAs content of the PL fraction corresponded to 18.5% and 18.1% for *U. armoricana* and *S. chordalis*, respectively. The main PUFAs in *U. armoricana* PL were 16:4*n-*3 (2%), 18:4*n-*3 (3.3%), 18:3*n-*3 (7.6%) and 18:2*n-*6 (1.7%). In addition, AA, EPA, and DHA acids were also detected as minor constituents. In the case of *S. chordalis*, 20:4*n-*6 and 20:5*n-*3 acids were the major PUFAs (12.2% and 3%, respectively). Their contents are higher than in edible red seaweeds such as *Chondrus crispus* or *Gracilaria verrucosa* and commercial fish oils [22].

Regarding the *n-*6/*n-*3 ratio, it was established at 0.1 for *U. armoricana* and 1 for *S. chordalis*. Five hydroxy FAs were identified in PL of *U. armoricana* representing 2.5%, including four 2-hydroxy FA (C_16_–C_22_) and one monounsaturated 3-hydroxy FA (C_17_). Three hydroxy FA were identified in PL of *S. chordalis* corresponding to 7.4%, including 2-hydroxy FA (C_16_–C_17_), whose one monounsaturated 2-hydroxy acids (C_16_) (Table 5). To our knowledge, these two monounsaturated 2-hydroxy and 3-hydroxy FA are reported in seaweeds for the first time.

All the spectra of the 2-hydroxy FAME exhibited molecular ions and other diagnostic fragment ions such as the ions at *m*/*z* 90 arising from the McLafferty rearrangement (instead of the usual *m*/*z* 74 for FAME) and *m*/*z* 103, the ion [M-MeOH]^+^, and a relatively intense [M-COOMe]^+^ ion [25,28,50,51,52,53]. The mass spectra of their NAP derivatives showed the molecular ion peak and prominent peaks at *m*/*z* 98 and 100, and a base peak at *m*/*z* 129 (McLafferty, 113 + 16). 3-Hydroxy FA was identified since the mass spectra of methyl esters and NAP derivatives showed base peaks at *m*/*z* 103 and 142, respectively [28,52].

The 2- and 3-hydroxy FA are only minor constituents of PL FA but they are ubiquitous in nature. They have been reported in marine sponge lipids, which is a marine organism that has bacterial symbiosis [50,51], and in the red algae *Schizymenia dubyi* (Gigartinales) [52] and *G. turuturu* (Halymeniales) [53]. Matsumoto *et al.* reported that 3- and 2-hydroxy FA in microalgae may be used to classify algal species [54]. Bacteria are also recognized as an important source of the hydroxy FA in the natural environment [55], and the 2-hydroxy FA are known to occur in sulfate-reducing bacteria [56]. Several short-chains 3-hydroxy FA were reported as antifungal substances [57], demonstrating that 2- and 3-hydroxy FA are able to influence the membrane properties. For instance, at lower temperatures, some bacteria modify their membranous fatty acid composition by increasing the amount of 2- and 3-hydroxy FA for maintaining the functional homeoviscous state of their membrane [19]. Moreover, there are several examples of close associations between bacteria and algae [58,59].

In addition, it is the first time that monounsaturated 3-hydroxy C_17_ was identified in *U. armoricana*. Until now, the 3-hydroxy short-chain acids were known as typical bacterial FA. Thus, this hydroxy FA identified in *U. armoricana* could be of symbiotic origin.

Furthermore, unidentified aldehyde dimethylacetals (DMA), were detected at trace levels in the FA of PL of *U. armoricana*. Their mass spectra displaying the characteristic fragment ion *m*/*z* 75 ([(CH_3_O)_2_-CH]^+^) as the base peak [51]. DMA revealed the presence of particular PL named plasmalogens known for various biological properties probably including protection against oxidation [60]. Such compounds have been reported from sponges and mollusks [51], and, very recently, in our previous study performed on *G. turuturu* [25].

In addition, variations in the FA composition can be attributed to environmental conditions, habitat, light, salinity, pollution, species and genetic status, location and seasonality, geography of development of the seaweed and to the method used for extracting oil [43]. Some recent investigations have demonstrated that FA profiles were specific to taxonomic groups [20,43,61,62]. Al-Hasan *et al.* have reported variations in macroalgae FA concentrations, but not in the composition pattern when the temperature varied [63]. According to Hotimchenko, light conditions influence the FA lipid contents and ratios [64].

In red seaweed, phosphatidylcholine and phosphatidylglycerol have been reported to be the major polar phospholipids, besides minor phosphatidylethanolamine, diphosphatidylglycerol and unidentified compounds [65].

#### 2.2.4. Fatty Acid Composition of Glycolipids

Glycolipids of *S. chordalis* were the main lipid group and the second for *U. armoricana*. The glycolipid composition from the two algae was analyzed by thin layer chromatography (TLC), as shown in Table 6. Both *U. armoricana* and *S. chordalis* were composed of monogalactosyldiacylglycerols (MGDG) (44% and 44.9%, respectively) digalactosyldiacylglycerols (DGDG) (28.8% and 23%, respectively), and sulfoquinovosyl diacylglycerols (SQDG) (27.2% and 32%, respectively).

**Table 6:** Glycolipids composition (% wt/wt) of *U. armoricana* and *S. chordalis*.

	*U. armoricana*	*S. chordalis*
MGDG ^a^	44.0	44.9
DGDG ^b^	28.8	23.0
SQDG ^c^	27.2	32.0

^a^ MGDG, monogalactosyldiacylglycerols; ^b^ DGDG, digalactosyldiglycerols; ^c^ SQDG, sulfoquinovosyl diacylglycerols.

The GL FAs of *U. armoricana* and *S. chordalis* were identified by GC-MS analyses as FAME and NAP. The FAs composition of GL is given in Table 7. More than 33 FAs were identified in GL of *U. armoricana* and more than 25 FAs in GL of *S. chordalis*, as shown in Table 7.

**Table 7:** Fatty acid (% of the glycolipids FAs mixture) of *U. armoricana* and *S. chordalis*.

Fatty Acid (FAs) ^a^	ECL ^c^	*U. armoricana* ^b^ (%)	*S. chordalis* ^b^ (%)
**Saturated fatty acids (SFAs)**			
12:0	12.00	0.04	nd ^b^
14:0	14.00	0.6	3.2
br-15:0	14.46	0.04	nd ^b^
*iso*-15:0	14.53	0.08	0.1
*ai*-15:0	14.63	0.5	nd ^b^
15:0	15.00	0.3	0.4
16:0	16.00	64.2	40.0
*ai*-17:0	16.72	0.5	nd ^b^
17:0	17.00	2.3	5.1
18:0	18.00	nd ^b^	3.1
19:0	19.00	1.2	9.9
20:0	20.00	0.2	0.1
21:0	21.00	2.4	nd ^b^
22:0	22.00	2.3	0.1
23	23.00	0.1	nd ^b^
24:0	24.00	0.5	0.7
**Total SFAs**		**75.2**	**62.7**
**Monounsaturated fatty acids (MUFAs)**			
13:1	12.72	1.5	nd ^b^
15:1	14.57	2.5	nd ^b^
16:1*n*-5	15.86	nd ^b^	4.1
16:1*n*-9	15.79	0.5	nd ^b^
16:1*n*-7	15.94	2.1	nd ^b^
17:1*n*-5	16.80	nd ^b^	15.4
17:1*n*-4	16.88	nd ^b^	0.5
18:1*n*-9	17.71	nd ^b^	3.6
18:1*n*-7	17.78	3.5	nd ^b^
18:1*n*-5	17.84	nd ^b^	1.6
19:1	18.86	nd ^b^	1.4
**Total MUFAs**		**10.1**	**26.6**
**Polyunsaturated fatty acids (PUFAs)**			
16:4*n*-3	15.61	2.1	nd ^b^
18:4*n*-3	17.70	0.2	nd ^b^
18:3*n*-3	17.77	3.5	nd ^b^
18:2	17.79	nd ^b^	0.2
18:2*n*-6	17.80	0.6	nd ^b^
18:2*n*-3	17.87	1.8	nd ^b^
20:5*n-*3	19.21	nd ^b^	1.2
20:4*n*-6	19.32	nd ^b^	2.1
22:6*n*-3	21.54	0.9	nd ^b^
22:3	21.83	nd ^b^	0.9
**Total PUFAs**		**9.0**	**4.5**
**Hydroxy fatty acids (Hydroxy FAs)**			
**2-Hydroxy FAs**			
2-OH-14:0	15.57	0.04	nd ^b^
2-OH-16:0	17.30	0.8	0.4
2-OH-18:0	19.23	0.2	2.7
2-OH-20:0	21.45	0.4	nd ^b^
2-OH-22:0	23.26	0.2	0.6
**3-Hydroxy FAs**			
3-OH-16:0	17.64	nd ^b^	0.5
3-OH-17:0	18.55	1.5	0.9
3-OH-18:0	19.46	0.3	nd ^b^
**Total Hydroxy FAs**		**3.5**	**5.1**
**Other compounds ^d^**		**2.2**	**0.7**
**Total *n*-6 PUFAs**		**0.6**	**2.1**
**Total *n*-3 PUFAs**		**8.5**	**1.2**
**Ratio *n*-6/*n*-3**		**0.07**	**1.7**

^a^ Methyl ester (%) of the glycolipids FAs mixture; ^b^ nd, not detected; ^c^ ECLs, equivalent chain lengths; *i*, *iso*; *ai*, *anteiso*; br, branched. Values are the mean of three replicates (mean % dw); ^d^ Mass spectra of the compounds showed characteristic intense fragment ion at *m*/*z* 75.

The SFAs represent 75.2% and 62.7% of the GL from *U. armoricana* and *S. chordalis*, respectively. They ranged from C_12_ to C_24_ for *U. armoricana* and from C_14_ to C_24_ for *S. chordalis*. The major SFA for *U. armoricana* was palmitic acid (64.2%). For *S. chordalis*, the main SFAs were myristic acid (3.2%), palmitic acid (40%), margaric acid (5.1%) and nonadecylic acid (9.9%) (Table 7).

Four branched-chain FA with short-chains were detected (br-15:0, *iso*-15:0, *ai*-15:0, and *ai*-17:0). Branched-chain FAs (*iso* and *anteiso*) are typical for the occurrence of bacteria and other monomethyl-branched short-chain FAs probably have the same origin [66]. Branched-chain FAs are characteristic of gram-positive bacteria [66].

MUFAs contents represented 10.1% and 26.6% of GL fraction for *U. armoricana* and *S. chordalis*, respectively. The main MUFA of *U. armoricana* were 15:1 (2.5%), 16:1*n-*7 (2.1%) and 18:1*n-*7 (3.5%). In the case of *S. chordalis*, the major MUFA were 16:1*n-*5 (4.1%), 17:1*n-*5 (15.4%) and 18:1*n-*9 (3.6%).

PUFAs content of the GL fraction corresponded to 9% and 4.5% for *U. armoricana* and *S. chordalis*, respectively. The main PUFAs in *U. armoricana* GL were 16:4*n-*3 (2.1%) and 18:3*n-*3 (3.5%). In addition, DHA acid was also detected as minor constituent. In the case of *S. chordalis*, 20:4*n-*6 and 20:5*n-*3 acids were the major PUFAs (2.1% and 1.2%, respectively).

Regarding the *n-*6/*n-*3 ratio, it was established at 0.07 for *U. armoricana* and 1.7 for *S. chordalis*.

Seven hydroxy FAs were identified in GL of *U. armoricana* at 2.2%, including five 2-hydroxy FA (C_14_–C_22_) and two 3-hydroxy FAs (C_17_–C_18_). Five hydroxy FA were identified in GL of *S. chordalis* at 0.7%, including three 2-hydroxy FA (C_16_–C_12_) and two 3-hydroxy acids (C_16_–C_18_). Moreover, it is the first time that 3-hydroxy short-chain acids were identified in *U. armoricana* and *S. chordalis*. Until now, the 3-hydroxy short-chain acids were known as typical bacterial FA. Thus, these hydroxy FA identified in *U. armoricana* and *S. chordalis* could be of symbiotic origin [50,51].

### 2.3. Antiproliferative Activity against Human Non-Small Cell Lung Cancer

This present study particularly focuses on the valorization of the major glycolipids, such as MGDG and DGDG. These lipids are reported to exhibit diverse biological functions, particularly antitumor activity, especially those from marine organisms.

The effect of MGDG and DGDG from *U. armoricana* and *S. chordalis* was evaluated for their capacity to inhibit *in vitro* the growth of human tumor cell Lines: NSCLC-N6 cell lines derived from a human non-small-cell bronchopulmonary carcinoma (moderately differentiated, rarely keratinized, classified as T2N0M0).

The results showed that only MGDG classes from *S. chordalis* were active against NSCLC-N6 cell lines with IC_50_ = 23.5 ± 1.3 µg/mL, and 24.0 ± 0.3 μg/mL for DGDG in *U. armoricana*.

Silva *et al.* showed that *Ulva rigida* and the red macroalga *Gelidium microdon* Kützing (Gelidiales), collected from the Azorean archipelago, exhibited *in vitro* growth inhibitory effect on human tumor cell lines: NCI-H460 (non-small cell lung cancer) [67]. The crude methanol extracts (after removal of chlorophylls) of both macroalgae were found moderately active against cell lines (IC_50_ = 42 µg/mL for *U. rigida* and IC_50_ = 65 µg/mL for *G. microdon*).

### 2.4. Proposed Algal Glycolipid Structures of Bioactive Constituents

To further identify the active constituents of *U. armoricana* and *S. chordalis* GL fraction, they were profiled by liquid chromatography coupled to high resolution mass spectrometry (HR-MS) [68]. The combination of the HR-MS data with the FA composition obtained by GC-MS of the active fraction was used to identify compounds responsible of the observed antitumor activity.

From the *U. armoricana*, the activity of the DGDG fraction was related to an ion *m*/*z* = 887.573 Da corresponding to the molecular formula of C_47_H_82_O_15_. This corresponded to a DGDG containing two Fas, 14:0 and 18:3*n*-3 (the major PUFA in DGDG). In the case of *S. chordalis* MGDG fraction, the antitumor activity was related to an ion *m*/*z* = 708.399 Da corresponding to the molecular formula of C_39_H_62_O_10_, the two FAs branched on the MGDG being 14:0 and 16:1*n*-5. In both cases the S1 and S2 position on the glycerol as well as the sugar moiety was undetermined.

## 3. Materials and Methods

### 3.1. Samples

*Ulva armoricana* (Ulvales, Chlorophyceae) was collected on the beach in Plestin-les-Grèves (48°39′28″ N, 3°37′47″ W), English Channel (Brittany, France), on 18 June 2012. *Solieria chordalis* (Rhodophyta, Gigartinales, Solieriaceae) was collected in October 2013 from the littoral zone of the Saint Gildas de Rhuys (47°30′0″ N, 2°49′60″ W, Atlantic coast, France). The algae were stored, and then thoroughly cleaned to remove epiphytes, sediment, organic debris, and macrofauna. Samples were successively rinsed with distilled water. The seaweeds were ground to pieces of about 3 mm with a hammer mill. The crushed seaweeds were frozen immediately at −25 °C and were thawed at the time of lipid analysis. The moisture content (%, MC) of seaweeds was determined by drying 2.00 g of samples in a thermo regulated incubator at 105 °C until constant weight and water content was determined gravimetrically. MC (%) = (*m*_i_ − *m*_od_/*m*_i_) × 100; with *m*_i_ = initial mass of wet seaweed specimen; *m*_od_ = oven dry mass of seaweed specimen.

### 3.2. Lipid Extraction, Lipid Classes, Fatty Acid and Sterol

Total lipids were extracted from fresh algae crushed (1 kg), with a mixture of chloroform/methanol (1:1, v/v) over 2 days at room temperature under agitation for 5 h. The extract was filtered using a Büchner funnel and washed with distilled water. The lipid content was determined by the gravimetric method and as a percentage of the algae dry weight.

The proportion of lipid relative to the dry mass corresponds to the weight ratio of the total lipid extract and biomass. The percentage (%) of lipid was calculated according to the following equation: % Fat = total lipid/(total lipid + dry mass without lipid) × 100. Mass of dry *S. chordalis* = 71.8 g; Mass of lipid content of *S. chordalis* = 2.2 g; Mass of dry *U. armoricana* = 88.6 g; Mass of lipid content of *U. armoricana* = 2.4 g.

One part of lipids (1 g) was fractionated into NL (dichloromethane), GL (acetone) and PL (methanol) by normal phase on flash column chromatography (SI-Std, 25 G, 50 μm, 22 bar, 20 mL/mn, IR-50SI/25G, Puri Flash^®^ Interchim, Montluçon, France). Fractions were evaporated to dryness and the percentage was determined as percentage of 1 g of lipids. Another part of lipids (50 mg) was saponified with 2 M ethanolic potassium hydroxide. A part of the unsaponifiable matter was acetylated using acetic anhydride and pyridine giving a mixture containing sterol acetates. The aqueous phase containing potassium salts of FA was acidified by 2 M HCl (pH = 4–5) and FA were extracted by dichloromethane. FAMEs were prepared by transmethylation (1 h at 80 °C with 6% methanolic hydrogen chloride). A part of these FAMEs was heated at 85 °C in a mixture of pyrrolidine and acetic acid for 1 h in order to obtain the *N*-acyl pyrrolidides (NAP). The FAMEs of GL were obtained by acidic methanolysis by heating 10 mg of GL with methanol/water/hydrochloric acid (29:4:3, v/v/v, 5 mL) at 80 °C for 18 h. The reaction mixture was extracted with water/hexane (3:9, v/v, 12 mL), the organic layer containing the FAME mixture was dried on anhydrous sodium sulfate, filtered and weighed after solvent evaporation. A part (1/3) of the FAME was preserved; the other was transformed into NAP, as described above.

Total FAs derivatives, PL FAs derivatives and GL FAs derivatives (FAME and NAP), sterols (as free forms and acetates), NL, were analyzed by gas chromatography coupled with mass spectrometry (GC-MS).

### 3.3. Thin Layer Chromatography

Thin layer chromatography (TLC) was carried out for the whole of sterols and glycolipids, in order to visualize the families of products. Thin layer chromatography was carried out on a plate of size 20 cm × 20 cm, consisted of an analytical polyester support and of a silica gel (60F254, 60 Å, 15 µm) of 0.25 mm thickness.

The sterols and unsaponifiable fractions composition were studied by TLC in addition to the analysis by GC-MS, using hexane, diethyl ether and acetic acid as eluent (85:15:0.1, v/v/v, double elution), with a standard cholesterol and cholesterol acetate, phytol, β carotene. Plates were visualized by spraying with sulfuric vanillin followed by heating in an oven.

As to the glycolipid class composition, the mobile phase was dichloromethane/methanol (85:15, v/v, double elution). In this case, standards used were monogalactosyldiglycerols (MGDG) (from spinach leaves and identified by nuclear magnetic resonance), digalactosyldiglycerols (DGDG), sulfoquinovosyl monoacylglycerols (SQMG) and sulfoquinovosyl diacylglycerols (SQDG) purchased from Sigma-Aldrich Co. (Saint-Quentin Fallavier, France). Plates were revealed by orcinol/sulfuric acid followed by heating in an oven.

### 3.4. Gas Chromatography-Mass Spectrometry Analyses of Fatty Acid and Sterol Derivatives

FAMEs, NAPs, sterols (as free forms and acetates), and neutral lipids were analyzed by GC-MS. The samples were analyzed using a Hewlett Packard 6890 series GC system coupled with a MS HP 6890 series, equipped with silica capillary column SLB™-5ms (60 m × 0.25 mm × 0.25 µm), the carrier gas was helium (1 mL·min^−1^). The analyses were carried out in electron impact (70 eV). Detector was set at 280 °C, and the injector at 250 °C. The samples were injected in splitless mode. Helium was used as the carrier gas under a constant flow rate (1 mL/min). Three different temperature gradients were used for FAME analysis, as follows: temperature was held at 170 °C for 4 min and programmed to 300 °C at 3 °C·min^−1^; for NAP analysis, 200 °C for 4 min then 3 °C·min^−1^ up to 310 °C and held for 20 min; and for sterols analysis, 200 °C then 3 °C·min^−1^ to 310 °C and held for 25 min. The solvent delay was 7 min for FAME and NAP analyses and 8 min for sterols.

The identification of FAs, sterols and unsaponifiable fractions is carried by identification their mass spectra. Thus, the FAs were identified as FAMEs by comparing their ECL values with those previously described or using commercial mixtures. The ECLs of the FAMEs were determined by expressing their elution positions relative to those of known straight-chain saturated FAME. The FAMEs were converted to *N*-acyl pyrrolidides in order to locate double bonds and branching.

### 3.5. Cellular Studies (NSCLC-N6)

The antiproliferative activity of glycolipids of *U. armoricana* and *S. chordalis* were evaluated on the NSCLC-N6 cell line [69] derived from a human non-small-cell bronchopulmonary carcinoma, moderately differentiated, rarely keratinized, classified as T2N0M0. The cell lines were cultured in RPMI 1640 medium with 5% fetal calf serum, to which were added 100 IU penicillin·mL^−1^, 100 mg streptomycin·mL^−1^ and 2 mM glutamine, at 37 °C in an air/carbon dioxide atmosphere (95:5, v/v).

Cytotoxicity was determined by continuous drug exposure. Experiments were performed in 96 wells microtiter plates (105 cells·mL^−1^ for NSCLC-N6). Cell growth was estimated by a colorimetric assay based on the conservation of tetrazolium dye (MTT) to a blue formazan product by live mitochondria [70]. Eight repeats were performed for each concentration. Control growth was estimated from 8 determinations. Optical density at 570 nm corresponding to solubilized formazan was read for each well on a Titertek Multiskan MKII. Experiments were performed at least in triplicate, 4 wells per glycolipid concentration being used. IC_50_ values were calculated from the dose-response curves.

### 3.6. Liquid Chromatography-Mass Spectrometry Analysis of Glycolipids

Active GL fractions were analyzed by high performance liquid chromatography coupled with an electrospray ionization ion trap time-of-flight multistage mass spectrometer (LCMS-IT-TOF) analyses to determine molecular formula. A Shimadzu LCMS-IT-TOF instrument composed of two LC-20ADxr pumps, a SIL-20ACxr autosampler, a CTO-20AC column oven, a SPD-M20A DAD detector, a CBM-20A system controller, an ESI ion source, and an IT-TOF mass spectrometer (Shimadzu, Kyoto, Japan) was used. Chromatographic conditions were adapted from Knittelfelder *et al.* [68]. LC-MS profiles were recorded with a 150 × 2.1 mm, 2.6 µm Kinetex C18 column (Phenomenex) in gradient mode at a flow rate of 0.4 mL·min^−1^ at the temperature of 60 °C. A solvent system composed of (A) water/MeOH 1:1 and (B) *iso*-propanol ; both eluant (A and B) being modified with 0.1% v/v formic acid and 10 mM of ammonium formate. The gradient consisted of 5 min at 25% B, followed by an increase to 45% B from 5 min to 13 min, followed by an increase to 75.27% B from 13 min to 45 min. The column was then washed with 100% *iso*-propanol for 7 min and re-equilibrated to 25% B for 7 min. injection consisted of 2 µL of solution at 1 mg/mL fraction in *iso*-propanol. The conditions of ESI-IT-TOF-MS analyses are listed below: (1) detection mode: positive ion and negative ion; (2) mass range: MS, *m*/*z* 100–1200 Da; (3) heat block: 250 °C and curved desolvation line temperature: 230 °C; nebulizing nitrogen gas flow: 1.5 L/min; interface voltage: (+) 4.0 kV, (−) 3.5 kV; detector voltage of the TOF analyzer: 1.61 kV; for MS the ion accumulation time: (+) 20 ms, (−) 5 ms; (4) All data were recorded and analyzed by Shimadzu software: LCMS solution Version 3.60, Formula Predictor Version 1.2, and Accurate Mass Calculator (Shimadzu, Kyoto, Japan); and (5) a trifluoroacetic acid sodium solution (2.5 mM) was used to calibrate the mass range from 100 to 1200 Da.

### 3.7. Statistical Analysis

All measurements were made in triplicate for each alga (*n* = 3), except for GL analyses, which were carried out on only one sample. All data are reported as mean ± standard deviation (SD). The statistical analysis was carried out on SPSS v20 (IBM, Chicago, IL, USA) using one-way analysis of variance (ANOVA).

## 4. Conclusions

The macroalgae *U. armoricana* and *S. chordalis* revealed low lipid contents. However, they exhibited high amounts of nutritionally essential *n-*6 and *n-*3 PUFAs, including EPA, AA, DHA, 16:4*n-*3, 18:4*n-*3, 18:3*n-*3, 18:2*n-*3, and 18:2*n-*6, but at lower levels than other edible red seaweeds such as *Chondrus crispus* or *Gracilaria verrucosa*. Therefore, *U. armoricana* and *S. chordalis* may be potential sources of *n-*3 and *n-*6 lipids. The health benefiting *n-*6/*n-*3 ratio in macroalgae allows their use in the formulation of functional foods and nutraceuticals. In this study, *U. armoricana* and *S. chordalis* can be considered as a source of dietary PUFAs, since they showed *n-*6/*n-*3 ratios ranging from 0.1 for *U. armoricana* to 1 for *S. chordalis*. Moreover, some FAs were identified for the first time in seaweeds as minor components such as 3-hydroxyoctadecanoic and the 2-hydroxy acid and two monounsaturated hydroxy acids. Hence, FAs compositions may provide a chemotaxonomic basis for macro-algae. These seaweeds contained interesting compounds such as phytol (precursor for the industrial synthesis of vitamins E and K), α-tocopherol (vitamin E) and squalene. Phytosterols were identified, namely brassicasterol, chondrillasterol, fucosterol and isofucosterol. The sterol composition showed also the presence of cholest-4-en-3-one. It would be of interest to isolate and identify the most important ones in terms of biological activities. Interestingly, glycolipids (MGDG, DGDG and SQDG) from *U. armoricana* and *S. chordalis* showed promising anti-proliferative activities on cancer cell lines.

## Abbreviations

AA: arachidonic acid
*ai*: *anteiso*
br: branched
DHA: *n*-3 docosahexaenoic acid
DGDG: digalactosyldiglycerols
DMA: dimethylacetal
dw: dry weight
ECL: equivalent chain lengths
EPA: *n*-3 eicosapentaenoic acid
FA_(s)_: fatty acid(s)
FAME_(s)_: fatty acid methyl ester_(s)_
fw: fresh weight
GC-MS: gas chromatography-mass spectrometry
GL: glycolipids
HR-MS: high resolution mass spectrometry
IC_50_: 50% inhibitory concentration
*I*: *iso*
LC-MS: liquid chromatography-mass spectrometry
LCMS-IT-TOF: liquid chromatography coupled with a electrospray ionization ion trap time-of-flight multistage mass spectrometer
la: lyophilized algae
MGDG: monogalactosyldiglycerols
MUFA_(s)_: monounsaturated fatty acids
NAP: *N-*acyl pyrrolidide_(s)_
NL: neutral lipids
PUFA_(s)_: polyunsaturated fatty acid(s)
PL: phospholipids
SFA_(s)_: saturated fatty acids
SQDG: sulfoquinovosyl diacylglycerols
SQMG: sulfoquinovosyl monoacylglycerols
s.d.: standard deviation
TL: total lipids
